# Pharmacogenomics implementation in cardiovascular disease in a highly diverse population: initial findings and lessons learned from a pilot study in United Arab Emirates

**DOI:** 10.1186/s40246-022-00417-9

**Published:** 2022-09-25

**Authors:** Zeina N. Al-Mahayri, Lubna Q. Khasawneh, Mais N. Alqasrawi, Sahar M. Altoum, Gohar Jamil, Sally Badawi, Dana Hamza, Lizy George, Anwar AlZaabi, Husam Ouda, Fatma Al-Maskari, Juma AlKaabi, George P. Patrinos, Bassam R. Ali

**Affiliations:** 1 Department of Genetics and Genomics, College of Medicine and Health Sciences, United Arab Emirates University, P.O. Box: 15551, Al-Ain, United Arab Emirates; 2 Department of Medicine, Tawam Hospital, Al-Ain, United Arab Emirates; 3 Nursing Department, Tawam Hospital, Al-Ain, United Arab Emirates; 4 The Heart Medical Center, Al-Ain, United Arab Emirates; 5 Zayed Centre for Health Sciences, United Arab Emirates University, Al-Ain, United Arab Emirates; 6 Public Health Institute, College of Medicine and Health Sciences, United Arab Emirates University, Al-Ain, United Arab Emirates; 7 Department of Internal Medicine, College of Medicine and Health Sciences, United Arab Emirates University, Al-Ain, United Arab Emirates; 8 School of Health Sciences, Department of Pharmacy, Laboratory of Pharmacogenomics and Individualized Therapy, University of Patras, Patras, Greece

**Keywords:** Pharmacogenomics, Pharmacogenomic-implementation, UAE, Precision medicine, Cardiovascular diseases, Clopidogrel, Statins, Warfarin

## Abstract

**Background:**

Pharmacogenomic (PGx) testing has proved its utility and cost-effectiveness for some commonly prescribed cardiovascular disease (CVD) medications. In addition, PGx-guided dosing guidelines are now available for multiple CVD drugs, including clopidogrel, warfarin, and statins. The United Arab Emirates (UAE) population is diverse and multiethnic, with over 150 nationalities residing in the country. PGx-testing is not part of the standard of care in most global healthcare settings, including the UAE healthcare system. The first pharmacogenomic implementation clinical study in CVD has been approved recently, but multiple considerations needed evaluation before commencing. The current report appraises the PGx-clinical implementation procedure and the potential benefits of pursuing PGx-implementation initiatives in the UAE with global implications.

**Methods:**

Patients prescribed one or more of the following drugs: clopidogrel, atorvastatin, rosuvastatin, and warfarin, were recruited. Genotyping selected genetic variants at genes interacting with the study drugs was performed by real-time PCR.

**Results:**

For the current pilot study, 160 patients were recruited. The genotypes and inferred haplotypes, diplotypes, and predicted phenotypes revealed that 11.9% of the participants were poor CYP2C19 metabolizers, 35% intermediate metabolizers, 28.1% normal metabolizers, and 25% rapid or ultrarapid metabolizers. Notably, 46.9% of our cohort should receive a recommendation to avoid using clopidogrel or consider an alternative medication. Regarding warfarin, only 20% of the participants exhibited reference alleles at *VKORC1*-1639G > A, CYP2C9*2, and CYP2C9*3, leaving 80% with alternative genotypes at any of the two genes that can be integrated into the warfarin dosing algorithms and can be used whenever the patient receives a warfarin prescription. For statins, 31.5% of patients carried at least one allele at the genotyped *SLCO1B1* variant (rs4149056), increasing their risk of developing myopathy. 96% of our cohort received at least one PGx-generated clinical recommendation for the studied drugs.

**Conclusion:**

The current pilot analysis verified the feasibility of PGx-testing and the unforeseen high frequencies of patients currently treated with suboptimal drug regimens, which may potentially benefit from PGx testing.

## Background

Pharmacogenomics (PGx) is a cornerstone in precision medicine practice. The last decades have witnessed considerable advances in PGx association discovery rates. In comparison, implementing these findings in clinical practice advanced at a slower pace [1]. Cost and reimbursement issues, educational and awareness challenges, and technical obstacles are among multiple challenges that hindered PGx findings' adoption in the clinic [2, 3]. However, drugs used in cardiovascular diseases (CVD) were among the leading PGx-implementations to prove significance and feasibility [2].

Indeed, translating PGx findings into clinical recommendations, like genetic-based dose adjustments or suggesting alternative medications, facilitates adoption by practitioners. This fact prompted the creation of treatment guidelines by professional societies, like the Clinical Pharmacogenetics Implementation Consortium (CPIC), the Dutch Pharmacogenetics Working Group (DPWG), and the Canadian Pharmacogenomics Network for Drug Safety (CPNDS). These organizations prioritized bridging the gap between PGx research and clinical practice by creating practical guidelines and updating them regularly according to the latest research findings. Drugs used in CVD, namely clopidogrel, warfarin, and statins, have PGx-dependent guidelines that address the personalization of their use [2, 3]. Moreover, PGx-implementation research provided solid and consistent evidence that supports the value of PGx-guided treatment with these specific agents [3].

Clopidogrel is an antiplatelet drug that belongs to the P2Y12 inhibitors group. It is a prodrug mainly metabolized to its active form by the CYP2C19 enzyme [4]. The impaired function enzyme produced by the loss-of-function (LOF) *CYP2C19* alleles interferes with the bioactivation of clopidogrel. Eventually, low active metabolite concentrations can put patients carrying these LOF alleles at a higher risk of thrombotic events [5, 6].

In comparison, warfarin-PGx associations include the pharmacogene encoding for its target protein, Vitamin K epoxide reductase complex 1 (*VKORC1*)*,* the (CYP2C9) enzyme responsible for its potent enantiomer metabolism, and another CYP enzyme, CYP4F2, interfering with vitamin K cycle. Variations in the three genes; *VKORC1*, *CYP2C9*, and *CYP4F2* (ordered according to their interaction significance magnitude), significantly affect the tolerated warfarin dose and can, together with clinical factors, explain almost 50% of interindividual variability [7, 8]. Conventionally, optimal warfarin-induced anticoagulation is measured by International Randomized Ratio (INR), which should be maintained between 2 and 3 in most indications [7]. Warfarin genetic dosing utilizes PGx-algorithms built on genetic testing results and clinical factors to suggest a starting dose of warfarin within 20% of the actual maintenance dose. IWPC and Gage are the most validated warfarin dosing PGx-algorithms [8]. Warfarin genetic dosing increases the time patient stays in the therapeutic INR range compared to the standard dosing [9].

In the case of statins, the PGx- association with the most robust evidence is *SLCO1B1* variants and statin-associated muscle symptoms (SAMS). *SLCO1B1* encodes for a solute carrier transport molecule involved in the liver intake of statins. Accordingly, this gene's variants, specifically rs4149056, can lead to increased exposure to statins outside the liver and have been repeatedly associated with SAMS [10, 11].

PGx-discovery research has been mainly carried out on Caucasian populations and demonstrates low diversity of participants and underrepresenting other ethnicities. This observation is aligned with the situation of genomic research in general [12]. Few efforts have been carried out to bridge this gap in the field of PGx [13, 14]. In comparison, scarce, if any, PGx-clinical implementation research has been carried out in populations other than those of Caucasian descent and, to a much lesser extent, people from East Asia [15].

United Arab Emirates (UAE) is a Middle Eastern country located in the Southeast part of the Arabian Peninsula, among other states of the Gulf Cooperation Council (GCC). The UAE’s current population of about 11 million is admixed, diverse, and multiethnic, with the majority being expatriates. CVDs are the first cause of mortalities in the country [16]. PGx-testing is not part of the standard of care in most UAE healthcare facilities for drugs used in CVDs. The first PGx-implementation research carried out on a large scale in the region has been launched lately. This study, designed as a randomized, multicenter, clinical PGx-implementation study, is planned to recruit 1,500 patients with CVDs or neurovascular diseases (NVDs) prescribed at least one of the following medications: clopidogrel, warfarin, atorvastatin, and rosuvastatin. This study is sought to illustrate the effect of PGx-testing on the patients’ outcomes and the healthcare system. Besides, it will evaluate the cost-effectiveness of PGx-testing practice upon its first introduction to the country. Establishing a biobank for patients with CVDs is also one of the planned secondary outcomes of this research.

Herein, there was a need to precede clinical implementation with a pilot study considering the country's unique population and healthcare system characteristics. The current report appraises the study design, testing, reporting systems, and the results of the pilot analysis. Through this pilot, we try to answer the following questions: in the diverse UAE population, are the actionable genetic variants selected from studies in other populations and known to interact with the medications chosen common and warrant an action? What is the optimal workflow for conducting on-demand PGx-testing without interfering with the healthcare procedures in an environment encountering these tests for the first time?

## Methods

### Study design and cohort recruitment

Adult patients (age > 18 years) prescribed at least one of the following selected drugs: anticoagulant warfarin, antiplatelet clopidogrel, and the cholesterol-lowering agents; atorvastatin and rosuvastatin were recruited for the current PGx pilot study. The recruitment settings involved two collaborating clinical sites: Tawam hospital and the Heart Medical Center in Al-Ain, UAE. Exclusion criteria of participants included potential patients that are (1) pregnant or breastfeeding, (2) having severe renal or hepatic impairment, and (3) with current active tumors or undergoing chemotherapy. After the completion of the consent process, blood samples and data were collected from all included subjects in compliance with the Declaration of Helsinki and following the “Abu Dhabi Health Research and Technology Ethical Committee” approval (DOH/CVDC/2020/1187).

### Data collection

For data collection, in-house electronic case report forms (eCRF) were created and integrated into the data capture system provided by Castor EDC [17]. A summary of the collected data is listed in Table 1. The collected data represent participants' clinical, lifestyle, and medical information at baseline, given that the same data will be collected in the extended study and for the CVD biobank. Nevertheless, other follow-up questionnaires were designed to gather more information at different time points for the extended research.

**Table 1:** Summary of the baseline collected information

Category	Collected information	Sub-information
Demographics	Gender	
	Year of Birth	
	Height (cm)	
	Weight (kg)	
	Highest completed education	
	Country of Origin	
	Ethnicity	
Health behavior	Smoking status: (Current, former, Never)	
	Smoking duration (days)	
	Smoking intensity (Packets/day)	
	Alcohol Consumption: (Current, former, Never)	
	Alcohol intensity (Units/week)	
	Physical activity (exercise times/week)	
Physical examination & signs at recruitment	Blood Pressure (mmHg)	
	Pulse (beats/min)	
Current diagnosis	Inpatient	Date of admission
		Reason of admission
		Recent interventional procedure
	Outpatient	Type of visit (first, follow-up, after intervention)
		Chief complaint
Study drugs	Which drug was the patient prescribed and recruited for (Clopidogrel, Warfarin, Atorvastatin, Rosuvastatin)	Start date
		Clinically recommended dose
Medical History	Major cardiac events	
	Neurovascular events	
	Other vascular events	
	Other morbidities	
	Surgery/intervention history	
Family History	Significant diseases in the family	
Concomitant medications	Check-box list of the medications available in the local market	
Laboratory Investigations	The most recent clinical laboratory investigations results (blood cholesterol, complete blood counts, and electrolytes)	

### DNA extraction

Whole blood samples from patients were collected in EDTA tubes. 500 μl of blood was lysed, and DNA was isolated using the Purelink™ Genomic DNA (Invitrogen), according to the manufacturer's protocol. Quantification and quality checks of DNA were then assessed using Nanophotometer (Implen NanoPhotometer®).

### Genotyping

Genotypes of *CYP2C19, VKORC1, CYP2C9, CYP4F2*, and *SLCO1B1* were determined using Taqman® SNP Genotyping Assays and Taqman® genotyping master-mix (Applied Biosystems, ThermoFisher Scientific). Given that there is scarce data about the common alleles of the selected genes in the study population, we selected the SNPs in the previous genes with the highest allele frequencies globally and those tagging actionable alleles which have published clinical recommendations. We have chosen to follow CPIC guidelines, given that these guidelines include recently updated versions for all the gene-drug pairs in this study. Table 2 lists the SNPs and associated star alleles on the studied pharmacogenes. Samples with already known genotypes were used as positive controls, and no-template controls (NTC) were used as negative controls. The genotyping experiments were carried out according to the manufacturer’s recommendations. The amplification quality was considered acceptable if it exceeded 95%.

**Table 2:** The studied drug-gene pairs and the tested variants

Affected Medication	Gene	SNP ID	Reference Allele	Alternative Allele
Clopidogrel	*CYP2C19*	rs4244285 (*2)	G	A
		rs12769205 (*2/*35)	G	A
		rs4986893 (*3)	A	G
		rs12248560 (*17)	C	T
		rs28399504 (*4)	A	G
		rs56337013 (*5)	C	T
		rs72552267 (*7)	A	G
		rs41291556 (*8)	C	T
Warfarin	*VKORC1*	rs9923231	C	T
	*CYP2C9*	rs1799853 (*2)	C	T
		rs1057910 (*3)	C	A
		rs28371686 (*5)	C	G
		rs9332131 (*6)	A	Del A
	*CYP4F2*	rs2108622	C	T
Statins	*SLCO1B1*	rs4149056	T	C

The genotypes of the studied pharmacogenes were translated into haplotypes and diplotypes and to metabolism phenotypes based on the haplotypes deposited in the central repository of pharmacogenes (PharmVar; https://www.pharmvar.org/), CPIC (https://cpicpgx.org/) annotations guidelines, and the information curated on the Pharmacogenomics Knowledgebase (PharmGKB https://www.pharmgkb.org/).

### Optimizing the pipeline

The pipeline needed optimization to ensure results delivery within 24 to 48 h of sample collection. Recruitment, taking patients' consent, and collecting blood samples are carried out between 7:30 a.m. and 12 p.m. on day zero. Sample transportation and DNA extraction are completed by 4 p.m. The genotyping is conducted the following day, and the results should be available by noon. Generated and reviewed reports can be sent to the treating physician before 4 p.m. on day one. In cases of genotyping failure, another genotyping run can be carried out between 12 p.m. and 4 p.m. on day one. Accordingly, the generated report will be delivered to the clinician in the early morning of day two before passing the 48 h window from recruitment.

### Statistical analysis

Descriptive statistics were calculated for patient characteristics, genotypes, inferred haplotypes, and diplotypes. Continuous variables were described as mean values, and standard deviations and categorical variables were summarized in percentages. The Chi-square test for independence was used to compare the number of minor allele carriers in our population to that reported in gnomAD all populations. The difference was considered significant if Chi-square test P-value was less than 0.01.

## Results

### Characterization of the included patients

For the current pilot study, we recruited 160 patients prescribed at least one of the study drugs. The participants' characteristics are listed in Table 3. Most (100/160, 62.5%) of the included patients were from the inpatient settings, while the rest were recruited from one cardiology outpatient clinic. The participants' mean age was 54 ± 14 years, ranging from 25 to 85. Most participants were males (130/160; 81.25%). Recruited patients descended from different ethnicities, where 52.5% were Arabs, 38% were South Asians, and 7.5% were Austronesians (from the Philippines and Indonesia).

**Table 3:** Participants’ characteristics

**Gender**	
Male	81(%)
Female	19(%)
**Age**	
Mean ± SD	55 ± 11 years
**Ethnicity**	
Arabs (Country)	52.5%
Egypt	13
Iraq	1
Jordan	17
Lebanon	4
Oman	6
Palastine	7
Sudan	7
Syria	11
UAE	14
Yemen	2
South Asians (Country)	38%
Afghanistan	4
Bangladesh	20
India	15
Nepal	1
Pakistan	23
Austronesians (Country)	7.5%
Indonesia	2
Philipines	8
Other (Country)	1.8%
Somalia	1
USA	1
UK	1

In the inpatient group (*N* = 100), 67% were starting on clopidogrel at recruitment. At the same time, 77 patients of all participants (*N* = 160, 48%) were prescribed clopidogrel at or before recruitment. Atorvastatin was the most commonly (119/160; 74.4%) prescribed drug among all participants. Rosuvastatin was prescribed less frequently (33/160; 21%), while warfarin was the least prescribed medication in our cohort (7/160; 4%). Moreover, 75/160 (47%) cases received a combination of clopidogrel and statin.

As expected, the clinical presentation of patients at recruitment varied widely between the inpatient and outpatient settings. In the inpatient group (*N* = 100), 64% of patients were admitted due to myocardial infarction (MI). In comparison, 17% were admitted due to stroke, and the rest were admitted for various reasons (e.g., atrial fibrillation, cardiomyopathy). In the same inpatients' group, 37% were recruited following a percutaneous coronary intervention in the. In comparison, 51 of the 60 participants (84%) in the outpatient group had a history of coronary artery disease, and seven patients (11%) had had at least one invasive cardiac intervention before recruitment. Patients in the outpatient group were mainly recruited because they were prescribed a statin; however, eight patients were also on clopidogrel, and two were using warfarin.

### PGx genotyping results

For *CYP2C19* tested variants, the alternative alleles at the two splicing variants, rs1276925 and rs4244285, were the most common, with frequencies of 26.6% and 25.3%, respectively. These alleles, defining together CYP2C19*2, appear with a frequency higher than their worldwide frequency in gnomAD [18] (17.4% and 17.95%, respectively) and the 1000Genome [19] (17.6% and 17.1%, respectively) databases. Another common alternative *CYP2C19* allele in our cohort is the CYP2C19*17 tag variant, rs12248560, which constituted 20% of the tested alleles, an equal frequency to that reported in gnomAD data (20%). Three SNPs (CYP2C19*5/*6/*8) showed no alternative alleles in our pilot cohort. The same variants are infrequent in all gnomAD populations [18].

Among all the tested variants, the alternative allele (T) in *VKORC1:* rs9923231 occurred with the highest frequency compared to all impaired function alleles in our cohort. It was reported with a frequency of 38%, which is higher than its frequency in all gnomAD populations (32.6%) [18]. Similarly, the impaired function allele C at *SLCO1B1*: rs4149056 was reported with a 16.9% frequency in our cohort, a frequency higher than in gnomAD and 1000Genome databases [19]. Table 4 lists the complete genotype frequencies at the tested variants in our cohort, with a comparison of the alternative alleles’ frequencies with their frequencies in gnomAD data (https://gnomad.broadinstitute.org/, accessed on 12 June 2022) and the results of the Chi-square test of independence. Statistically significant differences were found between the frequencies of alternative alleles in our cohort, and that reported in gnomAD all populations at the following variants: *CYP2C19*: rs4244285, rs12769205, rs4986893, rs12248560, *VKORC1*: rs9923231, and *CYP2C9*: rs1057910.

**Table 4:** Genotypes frequencies and a comparison of alternative allele frequency in UAE cohort and gnomAD all populations

Gene	SNP ID	Homozygous Ref	Heterozygous	Homozygous Alt	Alt. Allele freq.^1^	GnomAD Alt Allele freq	Chi-square statistic	*P*-value*
CYP2C19	rs4244285 (*2)	*92 (57.5%)*	*55 (34.4%)*	*13 (8.1%)*	*0.253*	*0.1749*	*13.54*	.00023**
	rs12769205 (*2/*35)	*91 (56.9%)*	*53 (33.1%)*	*16 (10%)*	*0.194*	*0.1795*	*16.07*	.000061**
	rs4986893 (*3)	*153 (95.6%)*	*7 (4.4%)*	*0*	*0.02*	*0.005*	*53.98*	< .00001**
	rs12248560 (*17)	*104 (65%)*	*48 (30%)*	*8 (5%)*	*0.2*	*0.205*	*375.41*	< .00001**
	rs28399504 (*4)	*160 (100%)*	*0*	*0*	*0*	*0.002*	*NA*	*NA*
	rs56337013 (*5)	*160 (100%)*	*0*	*0*	*0*	*0.00001*	*NA*	*NA*
	rs72552267 (*7)	*160 (100%)*	*0*	*0*	*0*	*0.0003*	*NA*	*NA*
	rs41291556 (*8)	*160 (100%)*	*0*	*0*	*0*	*0.001*	*NA*	*NA*
VKORC1	*rs9923231*	*66 (41.3%)*	*59 (36.9%)*	*38 (21.9%)*	*0.4*	*0.32*	*375.41*	< .00001**
CYP2C9	rs1799853 (*2)	*142 (88.8%)*	*13 (8.1%)*	*5 (3.1%)*	*0.072*	*0.09*	*1.41*	*.235*
	rs1057910 (*3)	*94 (58.8%)*	*64 (40%)*	*2 (1.3%)*	*0.213*	*0.06*	*125.8*	< .00001**
	rs28371686 (*5)	*160 (100%)*	*0*	*0*	*0.078*	*0.001*	*NA*	*NA*
	rs9332131 (*6)	*159 (99.4%)*	*1 (0.6%)*	*0*	*0.003*	*0.0009*	*1.534*	*.215*
CYP4F2	*rs2108622*	*80 (50%)*	*65 (40.6%)*	*15 (9.4%)*	*0.297*	*0.266*	*1.55*	*.213*
SLCO1B1	*rs4149056*	*110 (68.8%)*	*46 (28.8%)*	*4 (2.5%)*	*0.169*	*0.133*	*3.499*	*.061*

Italics indicates significance of Chi-square test

*Ref* Reference allele, *Alt* Alternative allele, *freq* Frequency, *NA* Testing is not applicable (one of the group counts equals zero)

^1^Frequencies of alternative alleles in the current cohort

^*^ *P*-value of Chi-square test

^**^Significant at *P* < .01

### Clinical impact and guiding therapy

The resultant genotypes were translated into haplotypes and diplotypes for *CYP2C19* and *CYP2C9*, according to the most recently updated haplotype definitions in PharmVar [19]. After inferring haplotypes and diplotypes, the phenotypes (enzyme activities) were predicted from the corresponding genes of the participants, as denoted in CPIC guidelines. Participants who were not found to carry any of the tested impaired function alleles were designated as “probably *1/*1” and predicted to have regular enzyme activity.

**Fig. 1:**
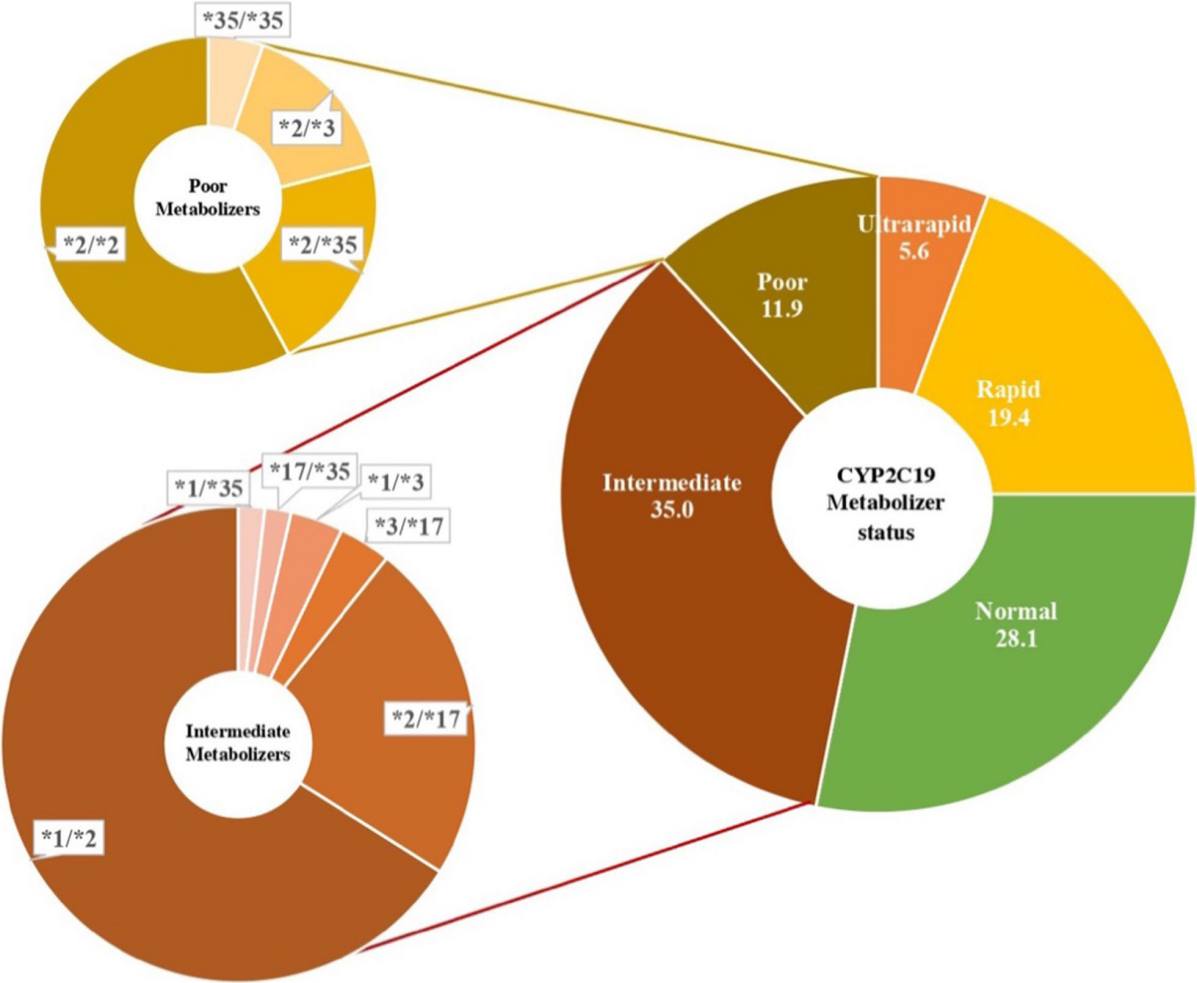
CYP2C19 diplotypes and predicted metabolizer status. Pie charts representing the CYP2C19 metabolizer status displayed in percentage and their associated diplotypes

For CYP2C19 enzyme activities, 19 participants (11.9%) were poor metabolizers (i.e., carry two of the impaired function alleles *2, *3, *4, *5, *6, *8, *35), 56 (35%) intermediate metabolizers (i.e., carry one of the impaired function alleles listed previously), 45 (28.1%) normal metabolizers, and 40 (25%) were rapid or ultrarapid metabolizers (i.e., have one or two *17 alleles, respectively). According to the latest CPIC issued CYP2C19-clopidogrel recommendations [20], patients with intermediate CYP2C19 activity should avoid clopidogrel if they are using it for acute coronary syndrome (ACS) or following a percutaneous cardiac intervention (PCI). Still, no similar recommendation is given for clopidogrel use in CVD indications other than ACS and PCI in intermediate metabolizers. In contrast, poor CYP2C19 metabolizers are recommended to avoid clopidogrel for all CVD indications. Moreover, those taking it for NVD indications, like strokes, should consider an alternative if they have an intermediate enzyme activity and avoid it if they have poor activity. Collectively, 46.9% of our cohort should receive a recommendation to avoid the use of clopidogrel or consider an alternative P2Y12 inhibitor. Figure 1 illustrates the distribution of our cohort according to their CYP2C19 metabolic status predicted by their genetic testing results.

**Fig. 2:**
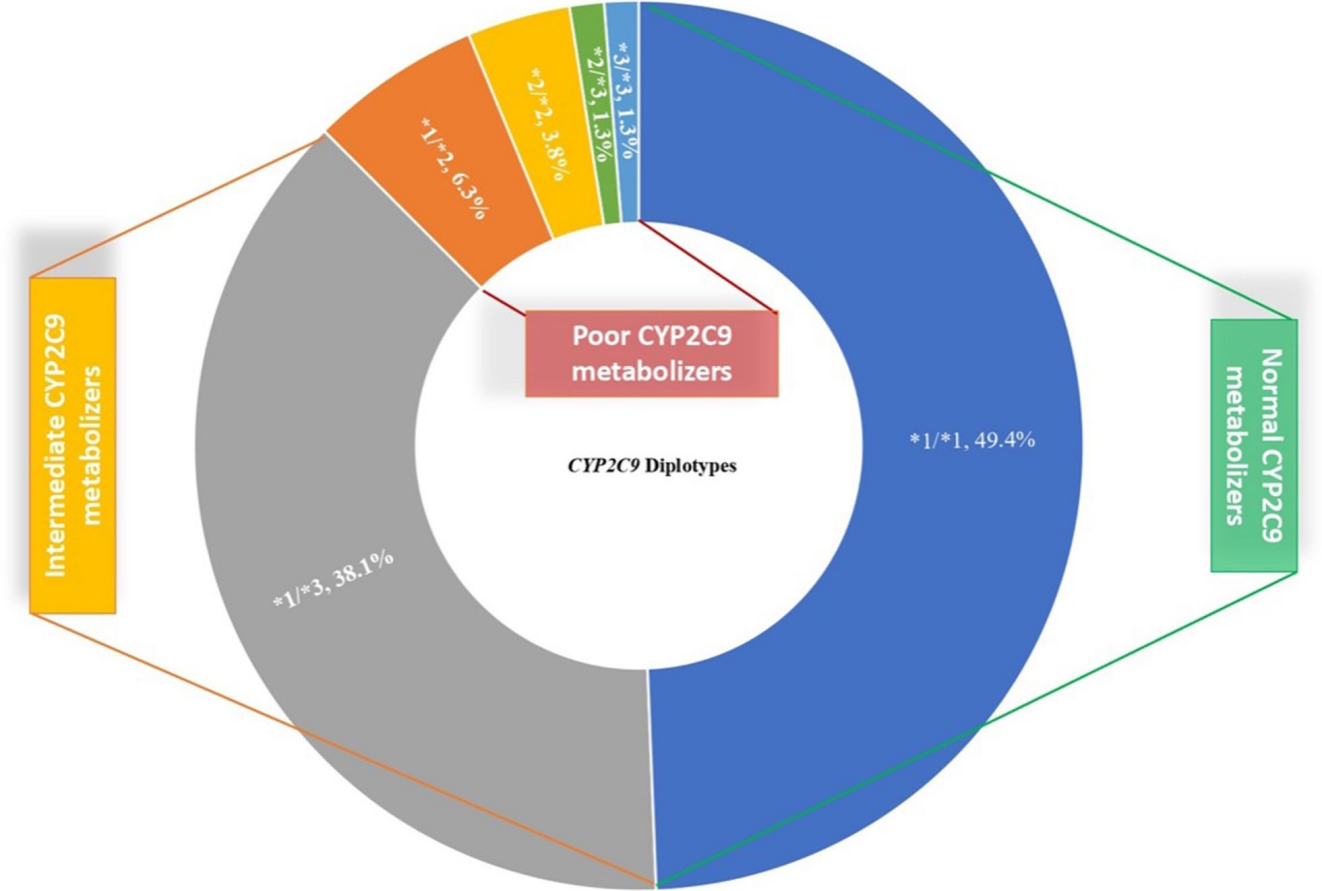
CYP2C9 diplotypes and predicted phenotypes. A pie chart demonstrates the predicted phenotypes and associated diplotypes of CYP2C9 pharmacogene

For *CYP2C9*, diplotypes were inferred from the most common star alleles, *2 and *3. In our cohort, 4% are poor *CYP2C9* metabolizers (i.e., *2/*2, *2/*3, or *3/*3), while 44.4% are intermediate metabolizers (i.e., one *2 or *3 allele). The results of *CYP2C9* diplotypes and predicted enzyme functionality are illustrated in Fig. 2.

**Fig. 3:**
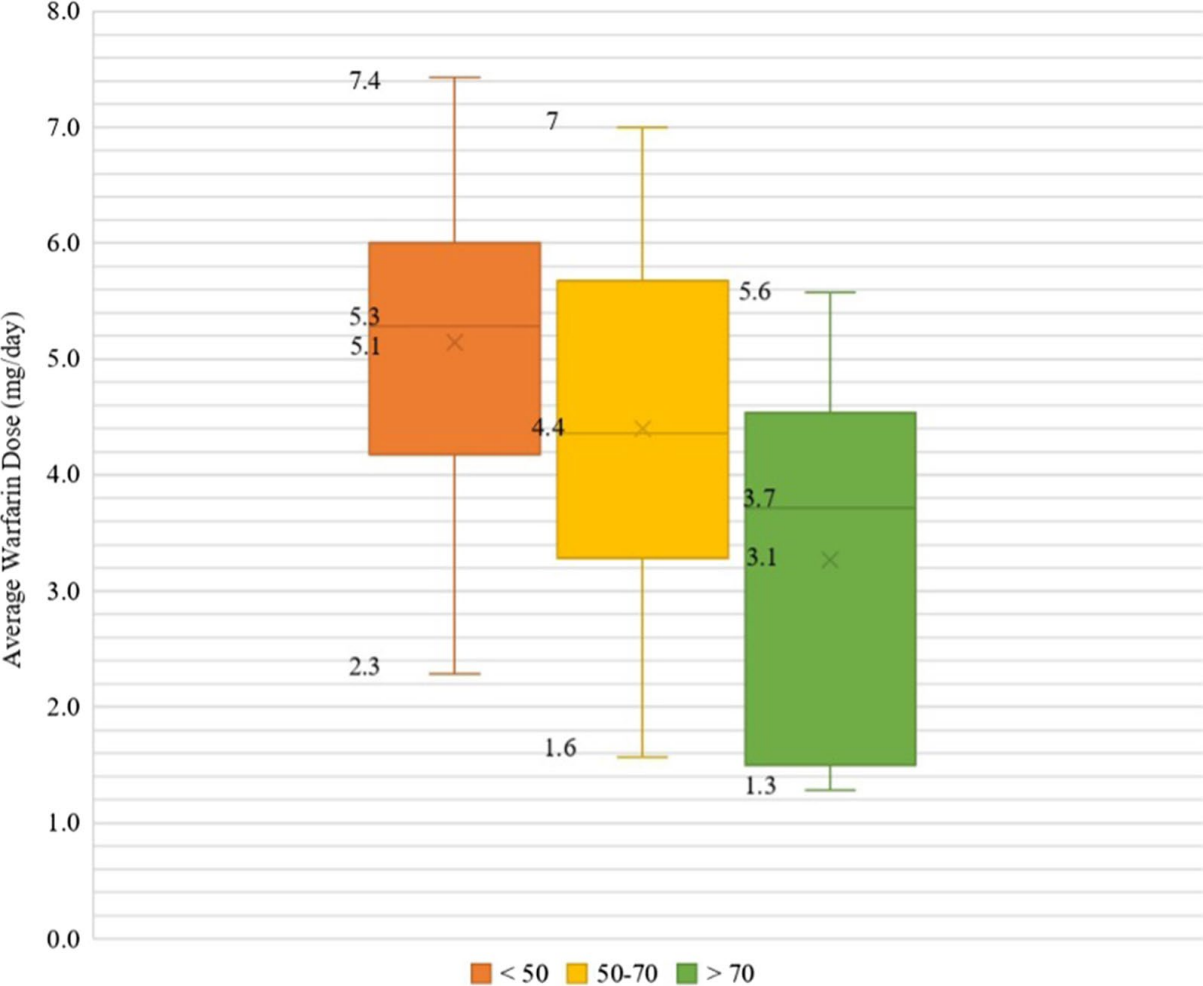
Average warfarin genetic dose for participants categorized by age. The genetic dose was calculated by the IWPC algorithm, which considers age, BMI, amiodarone use, using CYP2C9 inhibitors, and the genotypes at VKORC1-1639G > A, CYP2C9*2, and CYP2C9*3. The dose was adjusted for CYP4F2 rs2108622 genotypes and represented as mg/day. The numbers represent each group's minimum, maximum, median (the line), and average dose

Subsequently, we collectively analyzed variants in *CYP2C9* and *VKORC1*, which interact with warfarin doses. It was found that only 32 of the 160 participants (20%) carried wild-type (i.e., reference) alleles at *VKORC1-1639G* > *A*, CYP2C9*2, and CYP2C9*3, leaving 80% of our cohort with at least one alternative allele at any of the three SNPs. According to the CPIC warfarin pharmacogenetic-guided dosing guidelines [21], the presence of an alternative allele at any of these sites (*VKORC1*-1639G > A, CYP2C9*2, or CYP2C9*3) results in a strong recommendation for adjusting the warfarin dose according to one of the published dosing PGx-algorithms (e.g., IWPC algorithm [22]). Accordingly, if the patients’ genotypes are available in health records, 80% of our cohort are eligible for a genetic-modified warfarin dose whenever they need a warfarin prescription. Moreover, The same CPIC guidelines [21] recommend modifying the dose according to the presence of any of the following variants: CYP2C9*6/*8/*11 and *CYP4F2* rs2108622 T allele. Using the IWPC online available calculator [22], we calculated the genetic-warfarin dose of participants, then adjusted the resultant genetic dose for the other *CYP2C9* alleles and *CYP4F2* rs2108622 T allele. The participants were then grouped into three groups according to age, less than 50 years, 50 to 70 years, and more than 70 years. The range and average warfarin genetic dose for each group were calculated and represented as a box and whiskers plot in Fig. 3.

The resultant average dose for the three age groups was lower than that reported by Shendre and colleagues [23], who reported the average warfarin dose for the same three age groups from a real-world setting. The average warfarin dose was 8.1 mg/day compared to 5.1 mg/day in the young (< 50) group, 7.2 mg/day compared to 4.4 mg/day in middle-aged (50–70), and 7.2 mg/day compared to 3.1 mg/day for elderly (> 70), in Shendere and coworker’s cohort compared to ours, respectively. Nevertheless, we prefer interpreting these observed differences with extreme caution, given that we built our calculation on hypothesizing that our participants will be prescribed warfarin at the time of recruitment and utilized their recruitment age and BMI data, which are changing variables.

Indeed, warfarin was the least prescribed medication for our participants. We had only seven patients receiving a warfarin prescription at recruitment. Consequently, we tested their warfarin genotype-adjusted dose and compared it with their clinically prescribed one, which depended exclusively on the patient’s clinical characteristics. The result of this analysis is demonstrated in Table 5. Unfortunately, the observed warfarin dosing data are limited by this subset's low number of samples. However, the high prevalence of alternative *VKORC1* and *CYP2C9* alleles in the entire cohort indicates the potential significance of PGx-guided dosing in our population whenever warfarin is prescribed.

**Table 5:** Warfarin gene-calculated dose versus clinical-prescribed dose

Case	Age (years)	Height (meter)	Wight (Kilogram)	Genetic-calculated dose (IWPC algorithm) (mg/day)	Modified* genetic-calculated dose (mg/day)	Clinically prescribed starting dose (mg/day)
1	67	1.60	55	1.7	1.9	4
2	35	1.69	55.9	4.3	4.7	2.5
3	66	1.65	75	2.7	3.8	5
4	64	1.64	95.6	4.6	5.0	5
5	59	1.68	78	6.3	6.3	4
6	41	1.53	104	2.9	2.9	4.5
7	53	1.46	73	1.9	2.0	3

^*^Dose retrieved from the IWPC calculator after adjustment for *CYP2C9* rare alleles and *CYP4F2* variant

For statins, 50 participants (31.25%) carried at least one allele at the genotyped *SLCO1B1* variant, increasing their risk of developing myopathy. CPIC recently issued guidelines for statin-associated musculoskeletal symptoms depending on *SLCO1B1*, *ABCG2*, and *CYP2C9* [24]. Moreover, PharmVar recently published *SLCO1B1* haplotypes [25], which were cited in the new CPIC recommendations. Both updates were issued following the initiation of our pilot study and were not considered in its design. However, these updates prompted us to add new variants to our targeted SNPs in the extended clinical trial. The newly added variants will cover the most common *SLCO1B1* haplotypes (*5 and *15) and the *ABCG* actionable variant, rs2231142 G > T, which interacts with rosuvastatin.

Interestingly, when intersecting the recommendations generated, we found that only seven participants carried reference alleles at all the tested variants and received no recommendations in their PGx-testing reports. In other words, 96% (153/160) of participants received at least one PGx clinical guidance.

Finally, after optimizing the pipeline, pharmacogenomic testing results were issued within 24 to 48 h of collecting the samples. Ensuring that the clinician receives the reports within this time frame is crucial for the successful planning for the extended implementation trial.

## Discussion

The results of the current pilot study illustrate how common are the actionable genetic variants in our diverse population and provide evidence on the feasibility and potential benefits of applying on-demand PGx-testing in two different scenarios: inpatient and outpatient settings, in institutions naïve to the PGx-testing practice, and a population descending from diverse ethnicities.

Indeed, our current PGx-implementation initiative is thought to impact PGx-implementation practice locally and globally. Genotype results of a specific population broaden the knowledge of its genetics and create data that can be used in adjusting and prioritizing drug choices and healthcare strategies for the studied population [26]. Herein, our genotyping results, as listed in Table 4, illustrated significant differences in frequencies of impaired alleles in our population and the worldwide populations stated in the gnomAD database. Furthermore, at the global level, PGx studies in ethnically diverse populations are believed to increase the power of genetic discovery, strengthen the relevance of PGx-implementation in the clinic, and ensure the generalizability of findings [1].

Clopidogrel is a mainstay in antiplatelet therapy, considered the first choice in some cases [27]. We found that almost 47% of the participants, all suffering from at least one type of CVD or NVD, needed to be cautious or avoid using clopidogrel. Genotype-guided selection of antiplatelet therapy showed non-inferior protection compared to the standard-treatment group with other P2Y12 inhibitors in a large randomized controlled trial (*N* = 2488), though with fewer adverse events in the genotype-guided arm [28]. A real-world study from China confirmed the efficiency of returning *CYP2C19* genotype results in guiding P2Y12 choice among cardiologists [29]. Although our current study is not designed to test similar findings, as we did not return PGx results to the treating clinicians, the high percentage of patients with impaired *CYP2C19* alleles indicates the potential benefits of this implementation. The high rate of intermediate and poor clopidogrel metabolizers should alarm the practitioners in our region of the potential shortcomings of the conventional practice in clopidogrel prescription without considering the metabolic status of patients.

Regarding warfarin PGx-interactions, we found that 80% of our cohort can utilize their genotyping results to apply warfarin genetic dosing. A recent review highlighted that the current PGx evidence of warfarin does not reflect populations’ diversity and probably exacerbates health inequalities. The authors warrened that understudied populations exhibit different minor allele frequencies, an observation we reported here and in previous reports from our population [30], which threatens the applicability of warfarin dosing algorithms [31]. Herein, the planned inclusion of more clinical sites can recruit more patients to the warfarin group, which may enable further validation of genotype-guided warfarin dosing in our population. Oral anticoagulation stratification according to *CYP2C9* and *VKORC1* genotypes promises a cost-effective and clinical-effective strategy that cannot be achieved without further prospective testing [2].

We found that 32% of our cohort should receive a warning of a higher-than-normal risk of developing SAMS. Indeed, most PGx studies evaluated SAM-PGx associations and clinical utility related to simvastatin-induced SAMS [26, 27]. However, the latest CPIC recommendations [24] have considered data related to atorvastatin and rosuvastatin and provided clinical guidelines for the optimal gene-guided dosing for these drugs for current and prospective users. The tested variant, rs4149056 T > C, is part of SLCO1B1*5 and *15 alleles [25] and is believed to confer a three-fold increased risk of developing SAMS and explains more than 60% of these cases [32, 33]. Indeed, statins are among the most used drugs worldwide and in our population [34]. Our finding of a higher frequency of this risk allele in our population warrants an increased vigilance from practitioners to this understudied risk and its potential interference with the patient’s adherence to therapy and its effect on treatment outcomes [35].

One of the significant obstacles in PGx-testing adoption is the concern of integrating these tests within the clinic’s workflow [36, 37]. These concerns originate from variable barriers, including testing costs, lack of knowledge among healthcare providers, potential results misinterpretation, and uncertainty about how to incorporate PGx-testing within the clinical workflow [37]. According to an international survey conducted in 2019 [38], countries with high development index (HDI), of which UAE is one of them (https://hdr.undp.org/data-center/country-insights#/ranks), have the integration of PGx-testing with clinical workflow among the most significant challenges hindering its clinical adoption [38]. Herein, we critically appraised the procedure of on-demand PGx-testing in institutions naïve to this practice, besides emphasizing the potential benefits of putting this approach into practice.

Nevertheless, this study had several limitations. The recruitment was conducted in two sites in the same region, which imprecise generalizing the findings over the UAE population. However, our cohort represented the demographic distribution of the UAE population proportionally. The UAE population is dominated by males (70–75% of the population) (https://data.worldbank.org/). Also, the predominant ethnicities (South Asians, Austronesians, and Arabs) that compose the UAE population ( https://worldpopulationreview.com) were represented in our cohort. We also realize that the current sample size limits the power of this study. Besides, given the small number of each ethnic subgroup, we could not apply sub-analysis based on ethnicity. Similarly, the results related to warfarin should be considered with caution, given the limited sample size and the lack of studies evaluating the IWPC algorithm in our population. Despite these limitations, and given the absence of similar previous studies, the present report provided the needed evidence to initiate PGx-implementation in CVD on a larger scale.

## Conclusion

Introducing PGx-testing is frequently faced with concerns regarding the significance of its introduction into healthcare systems performance and the practicality of its application [39]. The current pilot analysis demonstrated the feasibility of PGx-testing and the unforeseen high frequencies of patients treated with suboptimal drug regimens, which may benefit from PGx testing.

Indeed, our findings prove that conducting explorative analysis, optimization, and procedure troubleshooting exercises are helpful before starting PGx-implementation initiatives and could provide favorable evidence for concerned practitioners.

## Data Availability

The datasets analyzed during the current study are not publicly available due to regulations of the local ethical committee but are available from the corresponding author upon reasonable request.
